# A New Multi-Attribute Emergency Decision-Making Algorithm Based on Intuitionistic Fuzzy Cross-Entropy and Comprehensive Grey Correlation Analysis

**DOI:** 10.3390/e22070768

**Published:** 2020-07-14

**Authors:** Ping Li, Ying Ji, Zhong Wu, Shao-Jian Qu

**Affiliations:** 1 Business School, University of Shanghai for Science and Technology, Shanghai 200000, China; liping185699744@163.com (P.L.); wuzhong@usst.edu.cn (Z.W.); 2 Management Engineering School, University of Nanjing for Information Science & Technology, Nanjing 210000, China; qushaojian@163.com

**Keywords:** multi-attribute emergency decision-making, intuitionistic fuzzy cross-entropy, grey correlation analysis, earthquake shelters, attribute weights

## Abstract

Intuitionistic fuzzy distance measurement is an effective method to study multi-attribute emergency decision-making (MAEDM) problems. Unfortunately, the traditional intuitionistic fuzzy distance measurement method cannot accurately reflect the difference between membership and non-membership data, where it is easy to cause information confusion. Therefore, from the intuitionistic fuzzy number (IFN), this paper constructs a decision-making model based on intuitionistic fuzzy cross-entropy and a comprehensive grey correlation analysis algorithm. For the MAEDM problems of completely unknown and partially known attribute weights, this method establishes a grey correlation analysis algorithm based on the objective evaluation value and subjective preference value of decision makers (DMs), which makes up for the shortcomings of traditional model information loss and greatly improves the accuracy of MAEDM. Finally, taking the Wenchuan Earthquake on May 12th 2008 as a case study, this paper constructs and solves the ranking problem of shelters. Through the sensitivity comparison analysis, when the grey resolution coefficient increases from 0.4 to 1.0, the ranking result of building shelters remains stable. Compared to the traditional intuitionistic fuzzy distance, this method is shown to be more reliable.

## 1. Introduction

At present, earthquakes, fires, novel coronavirus infections, and other frequent disasters have caused great loss to human beings. Owing to the uncertainty and fuzziness of such emergency problems, it is difficult for decision makers (DMs) to determine alternatives with real numbers to make quick decisions. The accurate processing of information has become an unavoidable problem in the development of the emergency decision [1,2,3] field. Under this urgent demand, fuzzy set theory, which can deal well with the uncertainty of decision-making problems, came into being [4]. Fuzzy sets [5,6] use membership as a single scale to reflect the support and opposition of DMs to objective things. However, with the development of decision theory, it is difficult to accurately describe the uncertainty of objective things by fuzzy sets alone. Based on this, Atanassov, a Bulgarian professor, put forward the concept of the intuitionistic fuzzy set (IFS) in the 1980s [7,8]. He used membership degree and non-membership degree to express the support, opposition, and hesitation of decision information. Compared to the fuzzy set, the IFS can describe the natural attributes of objective things more accurately [9,10,11].

The IFS is a new mathematical tool for dealing with uncertain and complex information efficiently, which is widely used in the field of multi-attribute decision-making (MADM) [12,13,14]. In recent years, scholars have made great progress in the research of intuitionistic fuzzy multi-attribute decision-making (IFMADM). The similarity measure is one of the most important decision-making methods in IFMADM. Xu et al. [15] systematically analyzed the similarity measurement formula based on geometric distance, set theory, and intuitionistic fuzzy matching degree. In order to improve the measurement accuracy of the similarity of the IFS, Park et al. [16] and Hu et al. [17] used the similarity measurement formula based on intuitionistic fuzzy entropy for the intuitionistic fuzzy number (IFN) and interval IFN, respectively, and optimized the alternatives. The IFS can represent the uncertainty of decision information well, but there are some difficulties in data comparison. Score function and precise function are effective means for data comparison and ranking in IFMADM. Chen et al. [18] were the first experts to study the score function of the IFN. They used the difference between membership and non-membership in the IFN to construct a function to compare the size relationship of the IFN, which is the basis of IFMADM. On the basis of score function, Hong et al. [19] proposed an intuitionistic fuzzy precise function, which greatly improved the efficiency of decision-making. The classical multi-attribute method has a wide range of development and application in the field of intuitionistic fuzzy. Table 1 summarizes some main methods of IFMADM.

Unfortunately, natural disasters, such as fires and floods, often lead to unexpected and disastrous consequences. A large number of emergency decision-making problems have evolved into MADM. Up to now, domestic and foreign scholars have conducted in-depth research in this field. Xu et al. [29] proposed a two-stage method to support the consensus-building process of large-scale MADMand applied it to earthquake shelter selection. Taking a fire and explosion accident as the study, Xu et al. [30] defined a generalized asymmetric language D number and proposed the corresponding MADM fusion algorithm, which verified the effectiveness of the method. Li et al. [31] proposed a risk decision analysis method based on the TODIM (an acronym in Portuguese of interactive and MADM) method to solve the emergency evacuation problem of tourist attractions, in which the attribute value and the probability of state occurrence are in the interval number format. This method solves this kind of emergency decision-making problem well, which shows that it is more effective than the traditional method. Based on an example of ship collision, Xiong et al. [32] used two intelligent algorithms, multi-attribute differential evolution algorithm and non-dominant sorting genetic algorithm, to verify the feasibility and effectiveness of the model. From the prediction model of the triple exponential smoothing method, Wang et al. [33] proposed an MADM additive weighting method, weighted product method, and elimination selection transformation reality method to sort the recycled electric vehicles, which provided an effective solution for managers and researchers in the electric vehicle industry and improved the efficiency of the electric vehicle industry. For the multi-attribute group decision-making problem of community sustainable development emergency response, Wu et al. [34] proposed a method based on subjective imprecise estimation of the reliability of binary language vocabulary, which greatly improved the efficiency of MADM. Karimi et al. [35] introduced the best and worst algorithm to solve the MADM problem in the fuzzy environment and applied this method to the evaluation of hospital maintenance, which proves the satisfactory performance of this method. Based on the above analysis, the MADM method is widely used in the field of emergency decision-making, which can solve the uncertainty well in the case of emergency. Table 2 summarizes some applications of the MADM method in emergency situations.

The above method is effective in solving the multi-attribute emergency decision-making (MAEDM) problem in a fuzzy environment. However, it has some limitations in the following aspects.

(1) In the case of emergency, DMs often have a certain subjective preference for alternatives, which is rarely studied.
(2) The traditional intuitionistic fuzzy distance measurement accuracy is not high. It is easy to have a situation where the IFN cannot be compared, which makes the decision result produce errors.
(3) For MAEDM problems with unknown or partially unknown attribute weights, the research is not deep enough and needs further analysis.
(4) There is no corresponding sensitivity analysis for the ranking results of alternatives, which fails to fully explain the reliability and stability of the evaluation mechanism.

According to the above limitations, the motivation of this paper is summarized as follows:

(1) With the increasing complexity of the global environment, many scholars focus on the field of emergency decision-making. Intuitionistic fuzzy multi-attribute emergency decision-making (IFMAEDM) is the focus of the current research.
(2) It is necessary to propose a distance measurement method based on the IFN, which can get rid of the shortcomings of traditional distance measurement and improve the reliability of decision results.
(3) The research on the uncertainty of attribute weight is the key problem in MAEDM. How to determine the weight is always the core of decision-making.
(4) The evaluation mechanism of the ranking results of alternatives can make the decision results more reliable.

Therefore, based on intuitionistic fuzzy and grey correlation analysis, this paper proposes a method to solve MAEDM by using intuitionistic fuzzy cross-entropy distance. First, the average information entropy of intuitionistic fuzzy is defined, and the measurement method of cross-entropy distance of intuitionistic fuzzy is given. On this basis, considering the unknown and known attribute weights, an optimization model with the subjective preference of the DMs is established and solved. Secondly, the intuitionistic fuzzy decision matrix is obtained according to the objective attribute evaluation of DMs. The intuitionistic fuzzy cross-entropy distance matrix is constructed by combining the objective evaluation value and subjective preference value of alternatives. Then, the attribute weight is determined according to the adjusted intuitionistic fuzzy average information entropy. By using the method of grey correlation analysis, the comprehensive grey relation coefficient of each alternative is obtained, and the order of alternatives is generated. Therefore, a new method is proposed to solve the MAEDM problem by using intuitionistic fuzzy cross-entropy and grey correlation analysis. The important contributions of this paper are mainly reflected in six aspects. (1) The intuitionistic fuzzy cross-entropy distance is defined. (2) A multi-attribute emergency decision with subjective preference is considered. (3) The uncertainty of attribute weight is discussed and solved by intuitionistic fuzzy information entropy. (4) The grey correlation analysis method is applied to MAEDM, which makes full use of decision-making information such as membership, non-membership, and hesitation. (5) According to the grey resolution coefficient, the sensitivity analysis is carried out to verify the reliability and stability of the decision results. (6) Compared to the traditional intuitionistic fuzzy distance, this method is shown to be more stable.

The remainder of this paper is organized as follows. Section 2 defines some basic knowledge of intuitionistic fuzzy theory and introduces the concept of intuitionistic fuzzy cross-entropy distance. In Section 3, a MAEDM model based on intuitionistic fuzzy cross-entropy and comprehensive grey correlation analysis is constructed. In Section 4, taking the ranking of earthquake shelters as an example, the practical application of this method is illustrated by comparing to the traditional intuitionistic fuzzy method. Lastly, Section 5 is the conclusion of the method proposed in this paper and the prospect of future research.

## 2. Preliminaries 

This section first reviews some basic concepts and definitions of intuitionistic fuzzy theory.

As the preference relationship in fuzzy theory is often assigned by the complementary 0.1–0.9 five-scale, we believe that the distribution of the levels between opposition and support is uniform and symmetric. However, in an actual situation, some problems require the use of a non-consistent and asymmetric distribution to evaluate variables, such as the marginal utility decline rate in economics. Therefore, it is very popular to solve this kind of asymmetric problem by fuzzy set theory.

**Definition** **1** **[4].** *If the domain*X*is a non-empty set, a fuzzy set is defined as:*

(1) $$A=\left\{<x,\mu_{A}(x)|x\in X\right\}$$

*which is characterized by a membership function*$\mu_{A}:X\to [0,1],$*where*$\mu_{A}\left(x\right)$*denotes the degree of membership of the element*x*to the set*A.

Ordinary fuzzy sets can only represent membership function, which refers to the support degree of an alternative without non-membership degree information. Therefore, Atanassov [7,8] extended the fuzzy set to the IFS. It is shown as follows:

**Definition** **2** **[7].** *If the domain*X*is a non-empty set, then the intuitionistic fuzzy set*A*on*X*can be expressed as:*

(2) $$A=\left\{<x,\mu_{A}(x),\nu_{A}(x)>|x\in X\right\}$$

*where*$\mu_{A}(x)$*and*$\nu_{A}(x)$*are the membership degree and non-membership degree of the element*x*belonging to*A*in the domain*X*, respectively,*

$$\begin{array}{l} \mu_{A}:X\to [0,1],x\in X\to \mu_{A}(x)\in [0,1]\\ \nu_{A}:X\to [0,1],x\in X\to \nu_{A}(x)\in [0,1], \end{array}$$

*It satisfies*$0\leq \mu_{A}(x)+v_{A}(x)\leq 1$; *let*

(3) $$\pi_{A}=1-\mu_{A}(x)-\nu_{A}(x)$$

*denote the degree of hesitation or uncertainty that element*x*in*X*belongs to IFS*A*,obviouslyfor any*x∈X*, with the condition*$0\leq \pi_{A}\leq 1$.

**Example** **1.** *Take an example to illustrate the specific meaning of the IFS. Suppose there is an IFS* A={<x,0.7,0.2>|x∈X} *, which indicates that the membership degree of IFS* X *is 0.7, the non-membership degree is 0.2, and the hesitation degree is 0.1. If we use this set to represent the voting process, assuming that the number of participants is 10, then 7 people support it, 2 oppose it, and 1 hesitates to remain neutral.*

**Definition** **3** **[36].** *Let*$\alpha_{A}=\left(\mu_{A},v_{A}\right)$*and*$\alpha_{B}=\left(\mu_{B},v_{B}\right)$*be the two intuitionistic fuzzy numbers. Then, the normalized Hamming distance between*$\alpha_{A}$*and*$\alpha_{B}$*is defined as follows:*

(4) $$d\left(\alpha_{A},\alpha_{B}\right)=\frac{1}{2}\left(\left|\mu_{A}-\mu_{B}\right|+\left|\nu_{A}-\nu_{B}\right|\right)$$

*where*$\mu_{A}\in \left[0,1\right]$*,*$v_{A}\in \left[0,1\right]$*and*$0\leq \mu_{A}+v_{A}\leq 1$*; meanwhile, all intuitionistic fuzzy numbers are expressed as*θ. *Obviously, the fuzzy number*$a^{+}=\left(1,0\right)$*is the maximum value in the fuzzy set, and*$a^{-}=\left(0,1\right)$*is the minimum value in the set.*

Geometric distance is not suitable for processing fuzzy decision information. According to the traditional distance model, Xu [15] proposed the distance measure formula of the intuitionistic fuzzy set:

**Definition** **4.** *Suppose*d*is a mapping:*d: ${\left(\phi \left(x\right)\right)}^{2}\to \left[0,1\right]$. *If there are intuitionistic fuzzy sets,*

$$\begin{array}{l} A=\left\{<x,\mu_{A}(x),\nu_{A}(x)>|x\in X\right\}\\ B=\left\{<x,\mu_{B}(x),\nu_{B}(x)>|x\in X\right\}\\ C=\left\{<x,\mu_{C}(x),\nu_{C}(x)>|x\in X\right\} \end{array},$$

*then the distance measure between the IFSs is*

(5) $$d_{Xu}={\left[\frac{1}{2n}\overset{n}{\underset{j=1}{\Sigma }}\left(\begin{array}{l} {\left|\mu_{A}(x_{j})-\mu_{B}(x_{j})\right|}^{\lambda }+{\left|\nu_{A}(x_{j})-\nu_{B}(x_{j})\right|}^{\lambda }\\ +{\left|\pi_{A}(x_{j})-\pi_{B}(x_{j})\right|}^{\lambda } \end{array}\right)\right]}^{\frac{1}{\lambda }}$$

*where*λ≥1*. When*λ=1*,*$d_{Xu}$*degenerates into Hamming distance with IFS:*

(6) $$d_{H}=\frac{1}{2n}\overset{n}{\underset{j=1}{\Sigma }}\left(\begin{array}{l} \left|\mu_{A}(x_{j})-\mu_{B}(x_{j})\right|+\left|\nu_{A}(x_{j})-\nu_{B}(x_{j})\right|\\ +\left|\pi_{A}(x_{j})-\pi_{B}(x_{j})\right| \end{array}\right)$$

*When*λ=2*,*$d_{Xu}$*degenerates into Euclidean distance with IFS:*

(7) $$d_{E}={\left[\frac{1}{2n}\overset{n}{\underset{j=1}{\Sigma }}\left(\begin{array}{l} {\left|\mu_{A}(x_{j})-\mu_{B}(x_{j})\right|}^{2}+{\left|\nu_{A}(x_{j})-\nu_{B}(x_{j})\right|}^{2}\\ +{\left|\pi_{A}(x_{j})-\pi_{B}(x_{j})\right|}^{2} \end{array}\right)\right]}^{\frac{1}{2}}$$

*Hamming and Euclidean distance formulas are an extension of intuitionistic fuzzy distance.*

Considering the attribute weight vector of $x_{j}\left(j=1,2,\ldots n\right)$, $\omega ={\left(\omega_{1},\omega_{2},\ldots ,\omega_{n}\right)}^{T}$, satisfies $0\leq \omega_{j}\leq 1$ and $\overset{n}{\underset{j=1}{\Sigma }}\omega_{j}=1$, and the above two distance formulas $d_{H}$ and $d_{E}$ can be expressed as:

(8) $$d_{H\omega }=\frac{1}{2n}\overset{n}{\underset{j=1}{\Sigma }}\omega_{j}\left(\begin{array}{l} \left|\mu_{A}(x_{j})-\mu_{B}(x_{j})\right|+\left|\nu_{A}(x_{j})-\nu_{B}(x_{j})\right|\\ +\left|\pi_{A}(x_{j})-\pi_{B}(x_{j})\right| \end{array}\right)$$

(9) $$d_{E\omega }={\left[\frac{1}{2n}\overset{n}{\underset{j=1}{\Sigma }}\omega_{j}\left(\begin{array}{l} {\left|\mu_{A}(x_{j})-\mu_{B}(x_{j})\right|}^{2}+{\left|\nu_{A}(x_{j})-\nu_{B}(x_{j})\right|}^{2}\\ +{\left|\pi_{A}(x_{j})-\pi_{B}(x_{j})\right|}^{2} \end{array}\right)\right]}^{\frac{1}{2}}$$

It is not difficult to see from the formula that all intuitionistic fuzzy distances satisfy the following properties:

(1) 0≤d(A,B)≤1;

(2) When A=B, d(A,B)=0

(3) d(A,B)=d(B,A);

(4) If A⊆B⊆C, d(A,B)≤d(A,C) and d(B,C)≤d(A,C).

In order to define the concept of intuitionistic fuzzy cross-entropy, the definition of information entropy is introduced. The average level of residual information after information redundancy eliminated is called information entropy, which is used to measure the uncertainty of information source in the communication process.

**Definition** **5.** *There is a discrete random variable* $X=\left\{x_{1},x_{2},\ldots ,x_{n}\right\}$ *that can be represented as:* $I=\left\{\begin{array}{l} x_{1},x_{2},\cdot \cdot \cdot ,x_{n}\\ p_{1},p_{2},\cdot \cdot \cdot ,p_{n} \end{array}\right\}$ *, where* $P=\left(p_{1},p_{2},\ldots ,p_{n}\right)$ *is the probability of discrete random variable* X *satisfying* $0\leq p_{j}\leq 1$ *and* $\overset{n}{\underset{i=1}{\Sigma }}p_{j}=1$ *; then, the information entropy of* I *can be expressed as*

(10) $$I=-\eta \overset{n}{\underset{j=1}{\Sigma }}p_{j}\log_{c}p_{j}$$

The constant η means the unit of measurement of information entropy, which is a constant greater than 0, and the base number c of the logarithmic function in the formula can take a non-negative constant. In particular, when c=2, the unit of information entropy is bit. When c=e, the unit of information entropy is nat. When c=10, its unit is dit. In general calculation, η=1, c=2.

Burillo et al. [37] extended the basic idea of information entropy to the field of intuitionistic fuzzy, and creatively used it to describe the uncertainty of the IFS.

**Definition** **6.** *Let* $X=\left\{x_{1},x_{2},\ldots x_{n}\right\}$ *be a domain and* $A=\left\{<x,\mu_{A}\left(x\right),v_{A}\left(x\right)>|x\in X\right\}$ *be an IFS on* X *. The intuitionistic fuzzy entropy of* A *can be expressed as:*

(11) $$E_{LH}(A)=\frac{1}{n}\overset{n}{\underset{i=1}{\Sigma }}\frac{1-\left|\mu_{A}\left(x_{i}\right)-\nu_{A}\left(x_{i}\right)\right|+\pi_{A}\left(x_{i}\right)}{1+\left|\mu_{A}\left(x_{i}\right)-\nu_{A}\left(x_{i}\right)\right|+\pi_{A}\left(x_{i}\right)}$$

**Definition** **7.** *Another equivalent transformation of intuitionistic fuzzy entropy* $E_{LH}$ *is:*

(12) $$E(A)=\frac{1}{n}\overset{n}{\underset{i=1}{\Sigma }}\frac{1-\max\left(\mu_{A}\left(x_{i}\right)-\nu_{A}\left(x_{i}\right)\right)}{1-\min\left(\mu_{A}\left(x_{i}\right)-\nu_{A}\left(x_{i}\right)\right)}.$$

**Proof.**  Model (11) and model (12) are equivalent.

$$\begin{array}{cc} E_{LH}(A) & =\frac{1}{n}\overset{n}{\underset{i=1}{\Sigma }}\frac{1-\left|\mu_{A}\left(x_{i}\right)-\nu_{A}\left(x_{i}\right)\right|+\pi_{A}\left(x_{i}\right)}{1+\left|\mu_{A}\left(x_{i}\right)-\nu_{A}\left(x_{i}\right)\right|+\pi_{A}\left(x_{i}\right)}\\ & =\frac{1}{n}\overset{n}{\underset{i=1}{\Sigma }}\frac{1-\left|\mu_{A}\left(x_{i}\right)-\nu_{A}\left(x_{i}\right)\right|+1-\mu_{A}\left(x_{i}\right)-\nu_{A}\left(x_{i}\right)}{1+\left|\mu_{A}\left(x_{i}\right)-\nu_{A}\left(x_{i}\right)\right|+1-\mu_{A}\left(x_{i}\right)-\nu_{A}\left(x_{i}\right)}\\ & =\frac{1}{n}\overset{n}{\underset{i=1}{\Sigma }}\frac{2-\left|\mu_{A}\left(x_{i}\right)-\nu_{A}\left(x_{i}\right)\right|-\left(\mu_{A}\left(x_{i}\right)+\nu_{A}\left(x_{i}\right)\right)}{2+\left|\mu_{A}\left(x_{i}\right)-\nu_{A}\left(x_{i}\right)\right|-\left(\mu_{A}\left(x_{i}\right)+\nu_{A}\left(x_{i}\right)\right)}\\ & =\frac{1}{n}\overset{n}{\underset{i=1}{\Sigma }}\frac{1-\frac{1}{2}\left(\left|\mu_{A}\left(x_{i}\right)-\nu_{A}\left(x_{i}\right)\right|+\left|\mu_{A}\left(x_{i}\right)+\nu_{A}\left(x_{i}\right)\right|\right)}{1-\frac{1}{2}\left(\left|\mu_{A}\left(x_{i}\right)+\nu_{A}\left(x_{i}\right)\right|-\left|\mu_{A}\left(x_{i}\right)-\nu_{A}\left(x_{i}\right)\right|\right)}\\ & =\frac{1}{n}\overset{n}{\underset{i=1}{\Sigma }}\frac{1-\max\left(\mu_{A}\left(x_{i}\right)-\nu_{A}\left(x_{i}\right)\right)}{1-\min\left(\mu_{A}\left(x_{i}\right)-\nu_{A}\left(x_{i}\right)\right)}=E(A) \end{array}.$$

□

Definition 7 is more concise in form and simpler in calculation. It eliminates the influence of hesitation and is a better expression of intuitionistic fuzzy entropy.

For the MAEDM problem discussed in this paper, when the attributes are completely unknown, it is necessary to calculate the average information entropy of each attribute. Combining with the intuitionistic fuzzy entropy, the intuitionistic fuzzy cross-entropy distance is defined as:

**Definition** **8.** *Suppose there is a domain*$X=\left\{x_{1},x_{2},\ldots ,x_{n}\right\}$*, where A and B are two IFSs on*X,

$$\begin{array}{l} A=\left\{<x_{j},\mu_{A}(x_{j}),\nu_{A}(x_{j})>|x_{j}\in X\right\}\\ B=\left\{<x_{j},\mu_{B}(x_{j}),\nu_{B}(x_{j})>|x_{j}\in X\right\} \end{array},$$

*then, the intuitionistic fuzzy cross-entropy distance formula of A andB is* [38]:

(13) $$\begin{array}{cc} CE\left(A,B\right)= & \overset{n}{\underset{j=1}{\Sigma }}\{\frac{1+\mu_{A}(x_{j})-\nu_{A}(x_{j})}{2}\times \\ & \log_{2}\frac{1+\mu_{A}(x_{j})-\nu_{A}(x_{j})}{1/2\left[1+\mu_{A}(x_{j})-\nu_{A}(x_{j})+1+\mu_{B}(x_{j})-\nu_{B}(x_{j})\right]}\}\\ & +\overset{n}{\underset{j=1}{\Sigma }}\{\frac{1-\mu_{A}(x_{j})+\nu_{A}(x_{j})}{2}\times \\ & \log_{2}\frac{1-\mu_{A}(x_{j})+\nu_{A}(x_{j})}{1/2\left[1-\mu_{A}(x_{j})+\nu_{A}(x_{j})+1-\mu_{B}(x_{j})+\nu_{B}(x_{j})\right]}\} \end{array}.$$

*As the intuitionistic fuzzy cross-entropy* CE(A,B) *does not satisfy the symmetry, considering the problems of emergency decision-making, let*

(14) $$CE^{*}\left(A,B\right)=CE\left(A,B\right)+CE\left(B,A\right)$$

*define the intuitionistic fuzzy cross-entropy distance combined with the characteristics of multi-attribute.*

**Theorem** **1.** *Referring to the properties of the intuitionistic fuzzy geometric distance formula, the intuitionistic fuzzy cross-entropy satisfies the following properties:*

(1) $0\leq CE^{*}\left(A,B\right)$;

(2) If A=B, $CE^{*}\left(A,B\right)=0$;

(3) If A⊆B⊆C, then $CE^{*}\left(A,B\right)\leq CE^{*}\left(A,C\right)$ and $CE^{*}\left(B,C\right)\leq CE^{*}\left(A,C\right)$.

**Proof.**  As

$$\begin{array}{cc} CE\left(A,B\right)= & \overset{n}{\underset{j=1}{\Sigma }}\{\frac{1+\mu_{A}(x_{j})-\nu_{A}(x_{j})}{2}\times \\ & \log_{2}\frac{1+\mu_{A}(x_{j})-\nu_{A}(x_{j})}{1/2\left[1+\mu_{A}(x_{j})-\nu_{A}(x_{j})+1+\mu_{B}(x_{j})-\nu_{B}(x_{j})\right]}\}\\ & +\overset{n}{\underset{j=1}{\Sigma }}\{\frac{1-\mu_{A}(x_{j})+\nu_{A}(x_{j})}{2}\times \\ & \log_{2}\frac{1-\mu_{A}(x_{j})+\nu_{A}(x_{j})}{1/2\left[1-\mu_{A}(x_{j})+\nu_{A}(x_{j})+1-\mu_{B}(x_{j})+\nu_{B}(x_{j})\right]}\} \end{array},$$

and model (13) has been given, the following exists

$$\begin{array}{cc} -CE\left(A,B\right)= & -\overset{n}{\underset{j=1}{\Sigma }}\{\frac{1+\mu_{A}(x_{j})-\nu_{A}(x_{j})}{2}\times \log_{2}\frac{1+\mu_{A}(x_{j})-\nu_{A}(x_{j})}{1/2\left[1+\mu_{A}(x_{j})-\nu_{A}(x_{j})+1+\mu_{B}(x_{j})-\nu_{B}(x_{j})\right]}\}\\ & +\overset{n}{\underset{j=1}{\Sigma }}\{\frac{1-\mu_{A}(x_{j})+\nu_{A}(x_{j})}{2}\times \log_{2}\frac{1-\mu_{A}(x_{j})+\nu_{A}(x_{j})}{1/2\left[1-\mu_{A}(x_{j})+\nu_{A}(x_{j})+1-\mu_{B}(x_{j})+\nu_{B}(x_{j})\right]}\}\\ & =\overset{n}{\underset{j=1}{\Sigma }}\{\frac{1+\mu_{A}(x_{j})-\nu_{A}(x_{j})}{2}\times \log_{2}\frac{1/2\left[1+\mu_{A}(x_{j})-\nu_{A}(x_{j})+1+\mu_{B}(x_{j})-\nu_{B}(x_{j})\right]}{1+\mu_{A}(x_{j})-\nu_{A}(x_{j})}\}\\ & +\overset{n}{\underset{j=1}{\Sigma }}\{\frac{1-\mu_{A}(x_{j})+\nu_{A}(x_{j})}{2}\times \log_{2}\frac{1/2\left[1-\mu_{A}(x_{j})+\nu_{A}(x_{j})+1-\mu_{B}(x_{j})+\nu_{B}(x_{j})\right]}{1-\mu_{A}(x_{j})+\nu_{A}(x_{j})}\} \end{array}$$

As the above logarithmic function is strictly convex, according to the relevant properties,

(15) $$\begin{array}{l} f\left(a_{1}x_{1}+a_{2}x_{2}+\ldots +a_{n}x_{n}\right)\leq \\ a_{1}f\left(x_{1}\right)+a_{2}f\left(x_{2}\right)+\ldots +a_{n}f\left(x_{n}\right) \end{array},$$

therefore, we can obtain the following expression,

$$\begin{array}{cc} -CE\left(A,B\right)\leq & \overset{n}{\underset{j=1}{\Sigma }}\log_{2}\{\frac{1+\mu_{A}(x_{j})-\nu_{A}(x_{j})}{2}\times \\ & \frac{1/2\left[1+\mu_{A}(x_{j})-\nu_{A}(x_{j})+1+\mu_{B}(x_{j})-\nu_{B}(x_{j})\right]}{1+\mu_{A}(x_{j})-\nu_{A}(x_{j})}\}\\ & +\overset{n}{\underset{j=1}{\Sigma }}\log_{2}\{\frac{1-\mu_{A}(x_{j})+\nu_{A}(x_{j})}{2}\times \\ & \frac{1/2\left[1-\mu_{A}(x_{j})+\nu_{A}(x_{j})+1-\mu_{B}(x_{j})+\nu_{B}(x_{j})\right]}{1-\mu_{A}(x_{j})+\nu_{A}(x_{j})}\}\\ \leq & \log_{2}\{[\left(1+\mu_{A}(x_{j})-\nu_{A}(x_{j})\right)+\left(1-\mu_{B}(x_{j})+\nu_{B}(x_{j})\right)+\\ & \left(1+\mu_{B}(x_{j})-\nu_{B}(x_{j})\right)+\left(1-\mu_{A}(x_{j})+\nu_{A}(x_{j})\right)]/4\}=0 \end{array}$$

Through the above proof, obviously, CE(A,B)≥0 and CE(B,A)≥0, and the same can be obtained. According to model (13) and (14), we can prove that $CE^{*}\left(A,B\right)\geq 0$. □

**Proof.**  When A=B, there are the following relationships: $\mu_{A}\left(x_{j}\right)=\mu_{B}\left(x_{j}\right)$,$v_{A}\left(x_{j}\right)=v_{B}\left(x_{j}\right)$. By substituting it into the model (13), we can obtain the conclusion CE(A,B)=0, CE(B,A)=0. Then, combining model (14), we can prove that $CE^{*}\left(A,B\right)=0$. □

**Proof.**  According to the understanding of the geometric intuitionistic fuzzy distance formula, it is not difficult to prove that the size of the fuzzy cross-entropy set is positively correlated with the size of distance. Let us assume that with A⊆B⊆C, we have $\mu_{A}\left(x_{i}\right)\leq \mu_{B}\left(x_{i}\right)\leq \mu_{C}\left(x_{i}\right)$ and $v_{A}\left(x_{i}\right)\leq v_{B}\left(x_{i}\right)\leq v_{C}\left(x_{i}\right)$. The following conclusions can be drawn: $\mu_{A}\left(x_{i}\right)-\nu_{A}\left(x_{i}\right)\leq \mu_{B}\left(x_{i}\right)-\nu_{B}\left(x_{i}\right)\leq \mu_{C}\left(x_{i}\right)-\nu_{C}\left(x_{i}\right)$. For the sake of proving convenience, $\mu_{A}\left(x_{i}\right)-v_{A}\left(x_{i}\right)$, $\mu_{B}\left(x_{i}\right)-v_{B}\left(x_{i}\right)$, and $\mu_{C}\left(x_{i}\right)-v_{C}\left(x_{i}\right)$ are recorded as a, b, c, respectively, and satisfy −1≤a≤b≤c≤1. Comparing the size relationship between two intuitionistic fuzzy cross-entropies can be done by subtraction. $\Delta CE^{*}=CE^{*}\left(A,C\right)-CE^{*}\left(A,B\right)$ can be transformed into:

$$\begin{array}{cc} \Delta CE^{*} & =\frac{1+a}{2}\log_{2}\frac{1+a}{1/2\left[1+a+1+c\right]}+\frac{1-a}{2}\log_{2}\frac{1-a}{1/2\left[1-a+1-c\right]}\\ & +\frac{1+c}{2}\log_{2}\frac{1+c}{1/2\left[1+c+1+a\right]}+\frac{1-c}{2}\log_{2}\frac{1-c}{1/2\left[1-c+1-a\right]}\\ & -\frac{1+a}{2}\log_{2}\frac{1+a}{1/2\left[1+a+1+b\right]}-\frac{1-a}{2}\log_{2}\frac{1-a}{1/2\left[1-a+1-b\right]}\\ & -\frac{1+b}{2}\log_{2}\frac{1+b}{1/2\left[1+b+1+a\right]}-\frac{1-b}{2}\log_{2}\frac{1-b}{1/2\left[1-b+1-a\right]} \end{array},$$

thus,

$$\begin{array}{cc} -\Delta CE^{*} & =\frac{1+a}{2}\log_{2}\frac{1/2\left[1+a+1+c\right]}{1+a}+\frac{1-a}{2}\log_{2}\frac{1/2\left[1-a+1-c\right]}{1-a}\\ & +\frac{1+c}{2}\log_{2}\frac{1/2\left[1+c+1+a\right]}{1+c}+\frac{1-c}{2}\log_{2}\frac{1/2\left[1-c+1-a\right]}{1-c}\\ & -\frac{1+a}{2}\log_{2}\frac{1/2\left[1+a+1+b\right]}{1+a}-\frac{1-a}{2}\log_{2}\frac{1/2\left[1-a+1-b\right]}{1-a}\\ & -\frac{1+b}{2}\log_{2}\frac{1/2\left[1+b+1+a\right]}{1+b}-\frac{1-b}{2}\log_{2}\frac{1/2\left[1-b+1-a\right]}{1-b} \end{array}.$$

As the $-\Delta CE^{*}$ is a strictly convex function, it has the property (15). It satisfies

$$\begin{array}{cc} -\Delta CE^{*} & \leq \log_{2}\left\{\begin{array}{l} \frac{1+a}{2}\times \frac{1/2\left(1+a+1+c\right)}{1+a}+\frac{1-a}{2}\times \frac{1/2\left(1-a+1-c\right)}{1-a}\\ +\frac{1+c}{2}\times \frac{1/2\left(1+c+1+a\right)}{1+c}+\frac{1-c}{2}\times \frac{1/2\left(1-c+1-a\right)}{1-c} \end{array}\right\}\\ & -\log_{2}\left\{\begin{array}{l} \frac{1+a}{2}\times \frac{1/2\left(1+a+1+b\right)}{1+a}-\frac{1-a}{2}\times \frac{1/2\left(1-a+1-b\right)}{1-a}\\ +\frac{1+b}{2}\times \frac{1/2\left(1+b+1+a\right)}{1+b}+\frac{1-b}{2}\times \frac{1/2\left(1-b+1-a\right)}{1-b} \end{array}\right\}=0 \end{array}.$$

Obviously, with $-\Delta CE^{*}\leq 0$, which is $\Delta CE^{*}\geq 0$, we can easily obtain $CE^{*}\left(A,C\right)\geq CE^{*}\left(A,B\right)$. The same reasoning can be proved, $CE^{*}\left(A,C\right)-CE^{*}\left(B,C\right)\geq 0$; thus, $CE^{*}\left(A,C\right)\geq CE^{*}\left(B,C\right)$. □

It can be seen from property (1) that the fuzzy entropy distance is non-negative. Property (2) means that when two IFSs are completely equal, the minimum intuitionistic fuzzy cross-entropy distance is equal to 0; thus, cross-entropy can be used to measure the difference degree or distance between two IFSs. Property (3) provides a sufficient basis for the comparison of intuitionistic fuzzy cross-entropy distance. Intuitionistic fuzzy cross-entropy extends the meaning of information entropy, which can be used to measure the fuzzy degree and unknown degree between IFSs on the basis of preserving the complete information of the original IFS. The greater the distance between two IFSs, the greater the cross-entropy of the fuzzy numbers. However, the traditional intuitionistic fuzzy distance measurement method cannot accurately reflect the differences between the data.

Based on this, a group of simple data can be used to compare the traditional intuitionistic fuzzy distance and fuzzy cross-entropy distance to show the reliability and stability of cross-entropy used to measure the degree of fuzzy.

**Example** **2.** *Suppose that there are three voting activities with a population of 10. The voting can be represented by three groups of fuzzy numbers:*$\alpha_{1}=\left(0.6,0.3\right)$*,*$\alpha_{2}=\left(0.5,0.4\right)$*,*$\alpha_{3}=\left(0.4,0.2\right)$. *First, we use the traditional Hamming and Euclidean distance model (6) and model (7), respectively, to solve*$d_{H}\left(\alpha_{1},\alpha_{3}\right)=d_{H}\left(\alpha_{2},\alpha_{3}\right)=0.3$*and*$d_{E}\left(\alpha_{1},\alpha_{3}\right)=d_{E}\left(\alpha_{2},\alpha_{3}\right)=0.2646$. *Obviously, it can be seen from the calculation results that two traditional distance formulas cannot measure the distance between fuzzy numbers*$\alpha_{1}$*and*$\alpha_{3}$*, or*$\alpha_{2}$*and*$\alpha_{3}$, *which is the disadvantage of the classical intuitionistic fuzzy distance measurement method. It is solved by the intuitionistic fuzzy cross-entropy distance method,*$CE^{*}\left(\alpha_{1},\alpha_{3}\right)=0.0037$*and*$CE^{*}\left(\alpha_{2},\alpha_{3}\right)=0.0101$.

The results show that the distance between $\alpha_{1}$ and $\alpha_{3}$ is closer than that of the traditional intuitionistic fuzzy distance. Therefore, it is more effective to introduce intuitionistic fuzzy cross-entropy to deal with uncertainty decision information. 

## 3. A Multi-Attribute Emergency Decision Model Based on Intuitionistic Fuzzy Cross-Entropy and Grey Correlation Analysis

This section analyzes the IFMAEDM problem in which DMs have a certain subjective preference for alternatives.

### 3.1. Problem Description

Taking the Wenchuan earthquake on May 12th 2008 as a study case, the government needs to build a batch of temporary shelters to rescue the victims in the disaster area. Considering the impact of earthquakes, the government has a certain priority (subjective preference) for the construction of regional shelters. After determining the geographical location, disaster risk, rescue facilities, and feasibility, a number of rescues in disaster-affected areas began in an orderly manner. The whole decision-making process aims to find the optimal solution through intuitionistic fuzzy cross-entropy and grey correlation analysis, which determines the area where the shelter is built first. It can be abstractly understood as: The decision-maker (government) gives the IFN representing the attribute value (agree, disagree, neutral) $\left(\mu_{ij},\nu_{ij}\right)$ from a series of alternatives (disaster-affected areas) $A_{i}\left(i=1,2,\ldots m\right)$ according to the objective evaluation attribute (specific factors of disaster situation) $C_{j}\left(j=1,2,\ldots n\right)$, which denotes that the decision maker’s approval degree is $\mu_{ij}$, objection degree is $\nu_{ij}$, and neutrality degree is $\pi_{ij}=1-\mu_{ij}-\nu_{ij}$ for alternative $A_{i}$ under the condition of attribute $C_{j}$. The attribute weight is expressed in $\omega_{j}$ and satisfies $0\leq \omega_{j}\left(j=1,2,\ldots n\right)\leq 1$ and $\overset{n}{\underset{j=1}{\Sigma }}\omega_{j}=1$. The IFN meets the following conditions: $0\leq \mu_{ij},\nu_{ij},\pi_{ij}\leq 1$. Using a fuzzy number to construct multi-attribute intuitionistic fuzzy decision matrix $R_{mn}$, the expression form is shown in Table 3:

Analyzing the Wenchuan earthquake, DMs have a certain subjective preference for alternatives, which need to consider the severity of the disaster area. The preference value is also IFN $c_{i}=\left(\sigma_{i},\delta_{i}\right)\left(i=1,2,\ldots m\right)$. The following content uses the method of intuitionistic fuzzy cross-entropy and grey correlation analysis to build the optimal decision model and solve it.

### 3.2. Steps of Intuitionistic Fuzzy Cross-Entropy and Grey Correlation Analysis Algorithm

**Figure 1:**
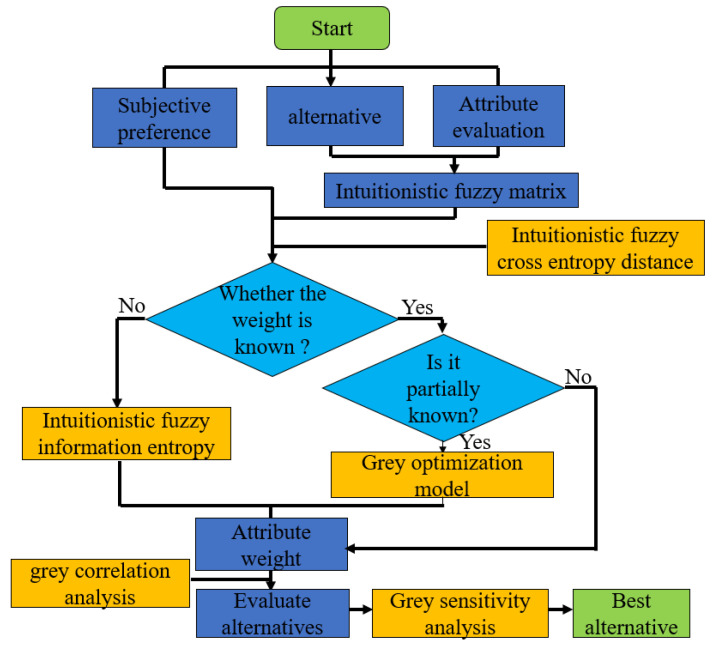
Algorithm framework of intuitionistic fuzzy cross-entropy and grey correlation analysis.

For the uncertain MAEDM problem with certain subjective preference, taking the Wenchuan earthquake shelter ranking problem for analysis, the comprehensive algorithm of intuitionistic fuzzy cross-entropy and grey correlation analysis is used to solve it. The specific steps are as follows (see Figure 1 for the flow framework):

**Step 1.** According to the data given in the background of the Wenchuan earthquake case, alternative $A_{i}$, objective evaluation attribute value $C_{j}$, decision maker’s subjective preference value $c_{i}$, and intuitionistic fuzzy evaluation decision matrix $R_{mn}$ are determined.

**Step 2.** Using intuitionistic fuzzy cross-entropy distance to solve the grey correlation coefficient between the objective evaluation value of alternatives and the subjective preference value of DMs, the formula is expressed as:

(16) $$\theta_{ij}=\frac{\underset{i}{\min}\;\underset{j}{\min}\;CE_{ij}^{*}+\xi \;\underset{i}{\max}\;\underset{j}{\max}\;CE_{ij}^{*}}{CE_{ij}^{*}+\xi \;\underset{i}{\max}\;\underset{j}{\max}\;CE_{ij}^{*}},$$

ξ is called the grey resolution coefficient, and the value range is 0≤ξ≤1, which is often set as ξ=0.5. It satisfies $0\leq \theta_{ij}\left(i=1,2,\ldots m;j=1,2,\ldots n\right)\leq 1$. The larger the grey correlation coefficient $\theta_{ij}$, the closer the objective evaluation value and subjective preference value. In model (16), $CE_{ij}^{*}$ is the intuitionistic fuzzy cross-entropy distance, and the specific formula is as follows:

(17) $$\begin{array}{cc} CE_{ij}^{*} & =\frac{1+\mu_{ij}-\nu_{ij}}{2}\times \log_{2}\frac{1+\mu_{ij}-\nu_{ij}}{1/2\left[1+\mu_{ij}-\nu_{ij}+1+\sigma_{i}-\delta_{i}\right]}\\ & +\frac{1-\mu_{ij}+\nu_{ij}}{2}\times \log_{2}\frac{1-\mu_{ij}+\nu_{ij}}{1/2\left[1-\mu_{ij}+\nu_{ij}+1-\sigma_{i}+\delta_{i}\right]}\\ & +\frac{1+\sigma_{i}-\delta_{i}}{2}\times \log_{2}\frac{1+\sigma_{i}-\delta_{i}}{1/2\left[1+\sigma_{i}-\delta_{i}+1+\mu_{ij}-\nu_{ij}\right]}\\ & +\frac{1-\sigma_{i}+\delta_{i}}{2}\times \log_{2}\frac{1-\sigma_{i}+\delta_{i}}{1/2\left[1-\sigma_{i}+\delta_{i}+1-\mu_{ij}{}_{i}+\nu_{ij}\right]} \end{array},$$

**Step 3.** On the basis of the solution method of the grey correlation coefficient given in model (16), the weight of each attribute is calculated to determine the comprehensive correlation coefficient $\theta_{i}$ of each alternative. The following three cases are discussed: The attribute weight is completely unknown, completely known, and the value range is known.

**Case 1.** Attribute weight is completely unknown. In order to determine the attribute weight, the average information entropy of each attribute must be obtained. On the basis of intuitionistic fuzzy entropy, the calculation method of information entropy is as follows:

(18) $$E\left(C_{j}\right)=-\frac{1}{\ln m}\overset{m}{\underset{i=1}{\Sigma }}\left(\frac{CE_{ij}^{*}}{\overset{m}{\underset{i=1}{\Sigma }}CE_{ij}^{*}}\ln\frac{CE_{ij}^{*}}{\overset{m}{\underset{i=1}{\Sigma }}CE_{ij}^{*}}\right),$$

The natural logarithm is taken to make the entropy value return to 1 and ensure the boundedness of information entropy. By transforming the formula of average information entropy, we can obtain the method of solving attribute weight:

(19) $$\omega_{j}=\frac{1-E\left(C_{j}\right)}{\overset{n}{\underset{k=1}{\Sigma }}\left[1-E\left(C_{k}\right)\right]}\left(j=1,2,\ldots n\right),$$

The weight parameters of each attribute can be determined and substituted,

(20) $$\theta_{i}=\overset{n}{\underset{j=1}{\Sigma }}\theta_{ij}\omega_{j}\left(i=1,2,\ldots m;j=1,2,\ldots n\right),$$

In model (20), the comprehensive correlation coefficient of alternatives $\theta_{i}$ can be aggregated.

**Case 2.** Attribute weights are fully known. Under the condition that the attribute is completely known, the grey correlation coefficient $\theta_{ij}$ of each alternative attribute is obtained by using model (16), and the comprehensive correlation degree $\theta_{i}$ of the alternative is obtained by combining model (20).

**Case 3.** The value range of attribute weight is known. Based on the maximum approach between weights with a known range of values and the subjective decision maker’s preference, a linear programming model with attribute weight as a variable is constructed,

(21) $$\begin{array}{l} \max\;Y\left(\omega_{j}\right)=\overset{m}{\underset{i=1}{\Sigma }}\overset{n}{\underset{j=1}{\Sigma }}\theta_{ij}\omega_{j}\left(j=1,2,\cdot \cdot \cdot n\right)\\ s.\;t.\left\{ \begin{array}{l} \overset{n}{\underset{j=1}{\Sigma }}\omega_{j}=1,\omega_{j}\in W\\ 0\leq \omega_{j},\left(j=1,2,\cdot \cdot \cdot n\right),\left(i=1,2,\cdot \cdot \cdot m\right) \end{array}\right. \end{array},$$

In this way, the weight parameters of each attribute can be determined.

The weight $\omega_{j}$ of each attribute can be calculated by establishing the optimization model of the maximum comprehensive grey correlation coefficient $\theta_{i}$:

(22) $$\theta_{i}=\overset{n}{\underset{j=1}{\Sigma }}\theta_{ij}\omega_{j}\left(i=1,2,\ldots m;j=1,2,\ldots n\right),$$

The corresponding linear programming model is constructed by programming software Matlab (R2017b) to solve the code, and the attribute weight of each alternative is obtained. Then, the model is substituted into (20) to determine the comprehensive correlation degree $\theta_{i}$.

**Step 4.** Based on the comprehensive correlation coefficient obtained under three different attribute weights in Step 3, the alternatives of the earthquake shelter are ranked according to the size relationship. The larger the $\theta_{i}$, the better the alternative, which is in the front row.

**Step 5.** The sensitivity analysis is made by setting different values of the grey resolution constant in the correlation coefficient, and the difference of ranking alternatives under different resolution coefficients is compared and analyzed.

## 4. A Numerical Case Study on the Ranking of Wenchuan Earthquake Shelters

In this section, the traditional intuitionistic fuzzy distance and the intuitionistic fuzzy cross-entropy distance are used to analyze and compare the ranking of earthquake shelters.

### 4.1. Intuitionistic Fuzzy Cross-Entropy Distance and Grey Correlation Analysis

The stability and reliability of the method of intuitionistic fuzzy cross-entropy and the grey correlation coefficient are analyzed through comparative experiments. Assume that the government carries out shelter assessment and optimization for the five areas with a large disaster impact, and use A, B, C, D, and E to represent them. The government analyzes and evaluates the geographical location $C_{1}$, disaster risk $C_{2}$, rescue facilities $C_{3}$, and feasibility $C_{4}$ of the five disaster areas. The decision-maker adopts an IFN to express the objective evaluation value of alternatives under different attributes, and the intuitionistic fuzzy decision matrix $R_{5\times 4}$ is shown in Table 4.

The decision maker’s subjective preference values for alternatives A, B, C, D, and E are also expressed by IFNs: $c_{1}=(0.5,0.4)$, $c_{2}=\left(0.6,0.3\right)$, $c_{3}=(0.4,0.3)$, $c_{4}=\left(0.4,0.5\right)$, and $c_{5}=\left(0.6,0.2\right)$. In order to choose the best alternative to build a shelter in the earthquake disaster area, the government adopts the intuitionistic fuzzy cross-entropy and grey correlation analysis method to make a decision.

**Step 1**. Determine the values of alternative A, B, C, D, and E; the objective evaluation attribute values $C_{1}$,$C_{2}$,$C_{3}$,$C_{4}$; the decision makers’ objective evaluation matrix $R_{5\times 4}$; and subjective preference values $c_{1}$,$c_{2}$,$c_{3}$,$c_{4}$,$c_{5}$.

**Step 2**. According to model (17), the intuitionistic fuzzy cross-entropy distance between the objective evaluation value and the subjective preference value of each alternative is calculated to form the distance matrix:

$$CE_{5\times 4}^{*}=\left[\begin{array}{l} 0.0000\;0.0151\;0.0000\;0.1378\\ 0.0151\;0.0038\;0.2402\;0.0411\\ 0.0000\;0.0327\;0.0348\;0.0151\\ 0.0036\;0.0000\;0.0036\;0.0145\\ 0.0041\;0.0159\;0.1810\;0.0041 \end{array}\right]$$

**Step 3**. Assuming that the grey resolution coefficient is ξ=0.5, the grey correlation coefficient between the decision-maker’s subjective preference value and the objective evaluation value is calculated according to model (16). The coefficient matrix is as follows:

$$\theta_{5\times 4}^{}=\left[\begin{array}{l} 1.0000\;0.8883\;1.0000\;0.4657\\ 0.8883\;0.9693\;0.3333\;0.7450\\ 1.0000\;0.7860\;0.7753\;0.8883\\ 0.9709\;1.0000\;0.9709\;0.8923\\ 0.9670\;0.8831\;0.3989\;0.9670 \end{array}\right]$$

**Step 4**. Calculate the attribute weight $\omega_{j}$ according to the known information provided by the above case. When the attribute weight is known, the model is relatively easy to solve. The following focuses on the analysis of two situations: The attribute weight is completely unknown and the attribute weight range is known.

**Case 1**. The weight of attributes is completely unknown. According to the idea of intuitionistic fuzzy entropy, the average intuitionistic fuzzy entropy of the attribute is obtained by combining model (18): $E\left(C_{1}\right)=0.5424$, $E\left(C_{2}\right)=0.7385$, $E\left(C_{3}\right)=0.5837$, $E\left(C_{4}\right)=0.6498$. Then, according to model (19), we obtain the attribute weight $\omega_{1}=0.3080$, $\omega_{2}=0.1761$, $\omega_{3}=0.2802$ and $\omega_{4}=0.2357$. The attribute weight obtained is substituted into model (22), and the comprehensive grey correlation coefficient $\theta_{i}$ of the alternatives under the attribute condition is calculated: $\theta_{1}=0.8544$, $\theta_{2}=0.7133$, $\theta_{3}=0.8730$, $\theta_{4}=0.9575$, and $\theta_{5}=0.7930$. From the comprehensive grey correlation coefficient $\theta_{i}$ of the alternatives, the result is $\theta_{4}>\theta_{3}>\theta_{1}>\theta_{5}>\theta_{2}$ and D≻C≻A≻E≻B. Therefore, the alternative D is the best and the government should give priority to building earthquake shelters in the region.

For proving the superiority and stability of the intuitionistic fuzzy cross-entropy and the comprehensive grey correlation analysis algorithm proposed in this paper, different resolution coefficients ξ are set for sensitivity analysis to compare and analyze whether the above alternatives will produce fluctuations. Set ξ=0.40, 0.50, 0.60, 0.70, 0.80, 0.90, 1.00. The results of the comprehensive correlation coefficient are shown in Table 5. The ranking results of alternatives did not fluctuate with the change in resolution coefficient.

**Figure 2:**
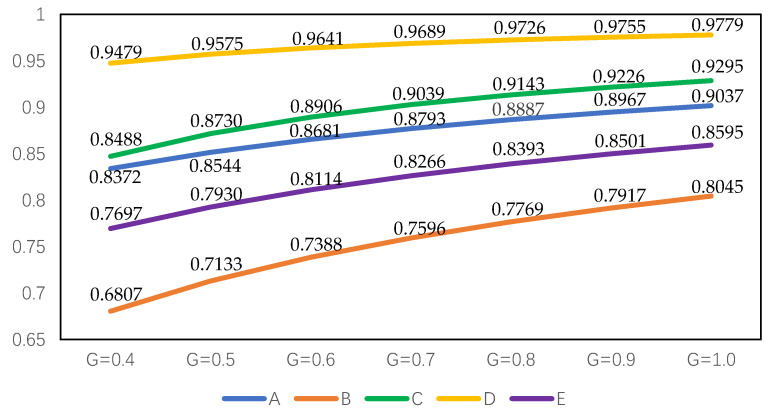
Ranking results of alternatives with different grey resolution coefficients based on completely unknown attribute weights.

In order to verify the reliability and stability of the method proposed in this paper more intuitively, we use Python graphics to carry out simulation experiments on the sequencing and gray resolution coefficient of each alternative, and the specific results are shown in Figure 2 (G is the grey resolution coefficient).

It can be seen from Figure 2 that in the seven experiments of sensitivity analysis of grey resolution coefficient, the ranking results of alternatives have not changed, and D≻C≻A≻E≻B is always maintained. The simulation experiment shows that D is the best alternative to build a shelter in the earthquake disaster area, and the decision result does not fluctuate, which shows the strong stability.

**Case 2**. The value range of attribute weight is known: $0.30\leq \omega_{1}\leq 0.32$, $0.17\leq \omega_{2}\leq 0.20$, $0.25\leq \omega_{3}\leq 0.28$, and $0.20\leq \omega_{4}\leq 0.24$. Through the linear programming model (21), the objective function Y to maximize the grey correlation coefficient of alternatives is constructed and solved:

(23) $$\begin{array}{cc} \max\;Y\left(\omega_{j}\right)= & 4.8262\omega_{1}+4.5267\omega_{2}\\ & +3.4784\omega_{3}+3.9583\omega_{4} \end{array},\;\begin{array}{c} s.\;t.\left\{ \begin{array}{l} 0.30\leq \omega_{1}\leq 0.32\\ 0.17\leq \omega_{2}\leq 0.20\\ 0.25\leq \omega_{3}\leq 0.28\\ 0.20\leq \omega_{4}\leq 0.24\\ \omega_{1}+\omega_{2}+\omega_{3}+\omega_{4}=1\\ 0\leq \omega_{j}\leq 1,\left(j=1,2,3,4\right) \end{array}\right. \end{array}$$

The attribute weight is $\omega_{1}=0.30$, $\omega_{2}=0.18$, $\omega_{3}=0.28$, and $\omega_{4}=0.24$ by MATLAB. Combined with model (22), the comprehensive grey correlation coefficient of each alternative is obtained: $\theta_{1}=0.8517$, $\theta_{2}=0.7131$, $\theta_{3}=0.8718$, $\theta_{4}=0.9573$, $\theta_{5}=0.7928$. According to the comprehensive grey correlation coefficient $\theta_{i}$ of the alternatives, we can obtain $\theta_{4}>\theta_{3}>\theta_{1}>\theta_{5}>\theta_{2}$. Therefore, the order of alternatives is D≻C≻A≻E≻B, and, thus, alternative D is the best. The government should give priority to building earthquake shelters in area D, which is the same as the decision-making result when the attribute weight is unknown.

In order to further verify the stability and superiority of the algorithm of intuitionistic fuzzy cross-entropy and comprehensive grey correlation analysis when the attribute weight range is known, different resolution coefficients are also set for sensitivity analysis, and the optimal alternative and decision results are compared. Taking ξ=0.40, 0.50, 0.60, 0.70, 0.80, 0.90, and 1.00, and attribute weight and comprehensive grey correlation analysis when the attribute weight range is known, different resolution coefficients are also set for sensitivity analysis, and the optimal alternative and decision results are compared. The attribute weight and comprehensive grey correlation coefficient of each alternative are shown in Table 6 and Table 7. From the table data, the change in the grey resolution coefficient does not affect the attribute weight and the decision-making result of the alternative, which is still D≻C≻A≻E≻B. It is always the best alternative to build the seismic shelter in the D area. In addition, when the weight is completely unknown, the comprehensive grey correlation coefficient of the alternatives is higher than that of the alternatives with known range of attribute weight. 

More importantly, when the grey resolution coefficient fluctuates from 0.4 to 1.0, whether the weight is known or unknown, the change range of the comprehensive grey correlation coefficient of alternative D is the smallest, which is 0.0300 and 0.0302, respectively (see Table 8). Alternative B is always the worst, and its fluctuation is also the largest, which is 0.1438 and 0.1239, respectively. Based on this, the stability of the proposed method is proved.

**Figure 3:**
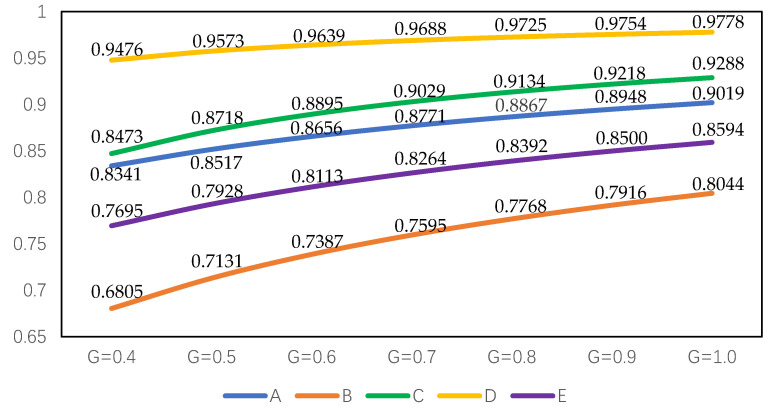
Ranking results of alternatives with different grey resolution coefficients based on known attribute weight range.

From Table 7, Python simulation results are shown in Figure 3. Compared to Figure 2, the comprehensive grey correlation coefficient decreases but does not change the overall trend of each alternative, and the decision results remain unchanged. Whether the attribute weights are known or not, the optimal alternative and ranking results are the same, which shows the superiority and stability of the method.

Through the above comparative analysis, the intuitionistic fuzzy entropy and grey correlation analysis method has achieved good results in solving the MAEDM problems. In this way, the ranking results have strong stability and environmental adaptability.

### 4.2. Traditional Intuitionistic Fuzzy Distance and Grey Correlation Analysis

Based on the data given by the above problem of ranking earthquake shelters, the traditional intuitionistic fuzzy distance and grey correlation degree are used to analyze and give the ranking results.

The traditional intuitionistic fuzzy distance model (4) has been given; thus, the corresponding grey correlation coefficient $\varepsilon_{ij}$ is

(24) $$\varepsilon_{ij}=\frac{\underset{i}{\min}\;\underset{j}{\min}\;d\left(r_{ij},c_{i}\right)+\xi \;\underset{i}{\max}\;\underset{j}{\max}\;d\left(r_{ij},c_{i}\right)}{d\left(r_{ij},c_{i}\right)+\xi \;\underset{i}{\max}\;\underset{j}{\max}\;d\left(r_{ij},c_{i}\right)}$$

where $r_{ij}$ denotes the objective evaluation value, $c_{i}$ denotes the subjective preference information, and grey resolution coefficient ξ=0.50. 

**Step 1**. Calculating the grey correlation coefficient of each alternative between the objective evaluation value and subjective preference information.

$$\varepsilon_{5\times 4}^{}=\left[\begin{array}{l} 0.6667\;0.6667\;1.0000\;0.4000\\ 0.6667\;0.8000\;0.3333\;0.5714\\ 1.0000\;0.5714\;0.5714\;0.6667\\ 0.8000\;1.0000\;0.8000\;0.6667\\ 0.8000\;0.6667\;0.8000\;0.8000 \end{array}\right]$$

**Step 2.** Determining the attribute weight. Due to the fact that the range of attribute weight values is known, utilize model (21) to establish the following single-objective programming model:

(25) $$\begin{array}{cc} \max\;Z\left(\omega_{j}\right)= & 3.9334\omega_{1}+3.7048\omega_{2}\\ & +3.5047\omega_{3}+3.1048\omega_{4} \end{array}\;\begin{array}{c} s.\;t.\left\{ \begin{array}{l} 0.30\leq \omega_{1}\leq 0.32\\ 0.17\leq \omega_{2}\leq 0.20\\ 0.25\leq \omega_{3}\leq 0.28\\ 0.20\leq \omega_{4}\leq 0.24\\ \omega_{1}+\omega_{2}+\omega_{3}+\omega_{4}=1\\ 0\leq \omega_{j}\leq 1,\left(j=1,2,3,4\right) \end{array}\right. \end{array}$$

Solving this model, attribute weight can be obtained:$\omega_{1}=0.30$, $\omega_{2}=0.18$, $\omega_{3}=0.28$, and $\omega_{4}=0.24$.

**Step 3.** On the basis of model (20), the comprehensive grey correlation coefficient is calculated: $\varepsilon_{1}=0.6960$, $\varepsilon_{2}=0.5745$, $\varepsilon_{3}=0.7229$, $\varepsilon_{4}=0.8040$, $\varepsilon_{5}=0.7760$.

**Step 4.** Determining the alternatives ranking. Rank the alternatives according to the size of the comprehensive grey correlation coefficient $\varepsilon_{i}$. Thus, D≻E≻C≻A≻B is the ranking result.

### 4.3. Comparative Analysis

Based on the ranking problem of earthquake shelters, this paper makes a comparative analysis from two aspects:

(1). The attribute weight is completely unknown and the attribute weight range is known

For a more intuitive comparison, it is further explored based on Figure 2 and Figure 3. Regardless of whether the attribute weight is known or unknown, the ranking results of alternatives maintain high stability. The best alternative is always D, and the worst is always B. The comprehensive grey correlation coefficient of the alternative is positively correlated with the grey resolution coefficient, which indicates that the larger the resolution coefficient, the greater the correlation coefficient of the corresponding alternative.

**Figure 4:**
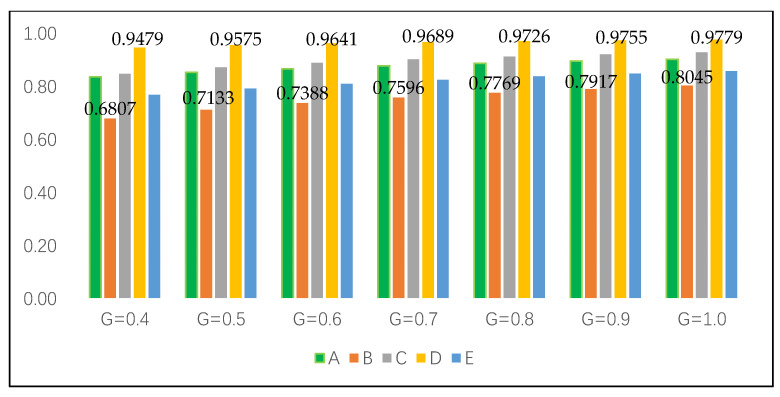
The alternatives with different grey resolution coefficients based on completely unknown attribute weights.

**Figure 5:**
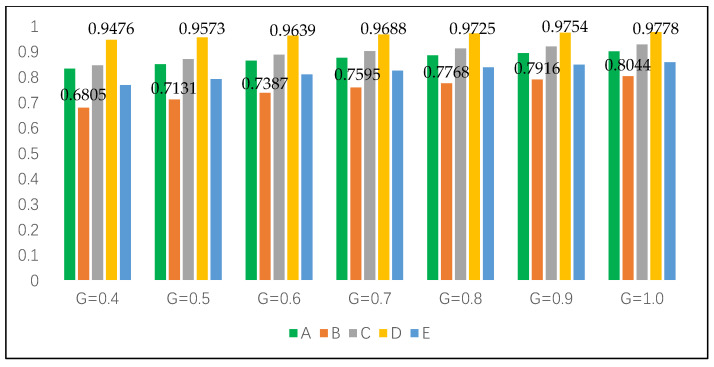
The alternatives with different grey resolution coefficients based on known attribute weight range.

Moreover, in the case of unknown weight, the comprehensive grey correlation coefficient of each alternative is always better than that of the known weight range, which also indirectly proves the fact that attribute weights are uncertain in most fields of decision problems (see Figure 4 and Figure 5). In addition, the results obtained by using a reasonable method to determine the attribute weights are more practical.

**Figure 6:**
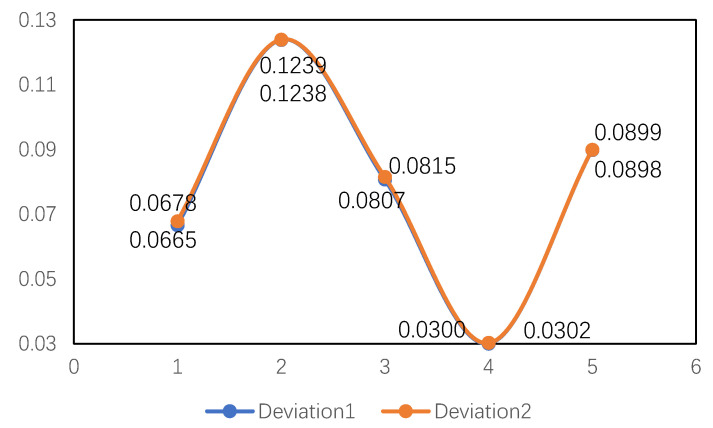
Deviation of comprehensive grey correlation coefficient in two cases.

Meanwhile, based on the data in Table 8, we can further analyze the volatility of the comprehensive grey correlation coefficient in two cases. From Figure 6 (deviation 1 represents unknown weights and deviation 2 represents known weights range), the deviation curves of the comprehensive grey correlation coefficient in the two kinds of weights situation almost coincide. However, when the weight is unknown, the fluctuation amplitude of the comprehensive grey correlation coefficient is still less than that of the known attribute weight range. 

Through the comparative analysis, we can see that the ranking result with unknown weight is more reasonable and more consistent with the uncertainty of the decision environment in MAEDM problems.

(2). The traditional intuitionistic fuzzy distance with the intuitionistic fuzzy cross-entropy distance

Through the above solution, the ranking results of the intuitionistic fuzzy cross-entropy method is D≻C≻A≻E≻B. Under the sufficient sensitivity analysis, the results maintain a high stability. However, by using the traditional intuitionistic fuzzy distance method, the result of ranking becomes D≻E≻C≻A≻B. Although the ranking result has little change, the best alternative is still D and the worst one is B (see Table 9). This also fully proves that the method based on intuitionistic fuzzy cross-entropy and grey correlation analysis proposed in this paper has strong stability.

According to the above two groups of comparative analysis, it can be concluded from many aspects that D is the best alternative. For the decision maker to make rescue measures, it is the most reasonable decision to give priority to the establishment of earthquake shelters in the D area.

## 5. Conclusions

This paper presents a new MAEDM method based on intuitionistic fuzzy cross-entropy and comprehensive grey correlation analysis. The main contributions are as follows: (1) Overcome the limitations of the traditional intuitionistic fuzzy geometric distance algorithm, and introduce the intuitionistic fuzzy cross-entropy distance measurement method, which can not only retain the integrity of decision information, but also directly reflect the differences between intuitionistic fuzzy data. (2) This paper focuses on the weight problem in MAEDM, and analyzes and compares the known and unknown attribute weights, which greatly improves the reliability and stability of decision-making results. (3) By using the method of grey correlation analysis, the fitting degree between the objective evaluation value and the subjective preference value of the decision maker can be fully considered. On this basis, a sensitivity analysis is made for the grey resolution coefficient to make the ranking result more reasonable. (4) The intuitionistic fuzzy cross-entropy and grey correlation analysis algorithm are introduced into the emergency decision-making problems such as the location ranking of shelters in earthquake disaster areas, which greatly reduces the risk of decision-making. (5) By comparing the traditional intuitionistic fuzzy distance to the intuitionistic fuzzy cross-entropy, the validity of the proposed method is verified.

Unfortunately, the method proposed in this paper is applicable to the emergency decision-making problems with certain subjective preference. For the emergency problems with which the decision maker has no obvious preference, the method needs to be further studied. In addition, considering more attribute indicators to rank alternatives may obtain more convincing results.

These aspects will become the research hotspot in the future: (1) In the MAEDM, the attribute weight problem will become a research focus. Considering the time factor, it may be an interesting topic to develop the weight into a dynamic field in the future. (2) The decision maker’s preference relation and attribute weight often have great uncertainty. It is an effective method to discuss the multi-attribute emergency decision by using a more reliable robust optimization [39,40,41].

## Figures and Tables

**Table 1:** A brief overview of preprocessing methods in intuitionistic fuzzy multi-attribute decision-making (IFMADM).

Literatures	Methods
Xu [15], Park et al. [16]	Similarity measure
Hu et al. [17]	Similarity measure, Fuzzy entropy
Chen et al. [18]	Score function
Hong et al. [19]	Intuitionistic fuzzy precise function
Wu et al. [20]	AHP, Score judgment matrix
Keshavarzfarda et al. [21]	AHP, DEMATEL
Chatterjee et al. [22], Liao et al. [23]	TOPSIS, VIKOR
Wu et al. [24], Vahdani et al. [25], Yu et al. [26]	ELECTRE, PROMETHEE
Meng et al. [27]	Prospect theory
Luo et al. [28]	Regret theory

**Table 2:** A brief literature list on the applications of multi-attribute decision-making (MADM) methods in emergency situations.

Literatures	Methods	Applications
Xu et al. [29]	Two-stage theory	Earthquake shelter selection
Xu et al. [30]	Generalized asymmetric language	Fire and explosion accident
Li et al. [31]	Risk decision analysis	Electric vehicle industry
Xiong et al. [32]	Evolution and non-dominant sorting genetic algorithm	Ship collision
Wang et al. [33]	Additive weighting	Electric vehicle industry
Wu et al. [34]	Subjective imprecise estimation of binary language	Community development
Karimi et al. [35]	The best and worst algorithm	Hospital maintenance

**Table 3:** Intuitionistic fuzzy decision matrix.

Alternative	$C_{1}$	$C_{2}$	…	$C_{n}$
$A_{1}$	$\left(\mu_{11},\nu_{11}\right)$	$\left(\mu_{12},\nu_{12}\right)$	…	$\left(\mu_{1n},\nu_{1n}\right)$
$A_{2}$	$\left(\mu_{21},\nu_{21}\right)$	$\left(\mu_{22},\nu_{22}\right)$	…	$\left(\mu_{2n},\nu_{2n}\right)$
…	…	…	…	…
$A_{m}$	$\left(\mu_{m1},\nu_{m1}\right)$	$\left(\mu_{m2},\nu_{m2}\right)$	…	$\left(\mu_{mn},\nu_{mn}\right)$

**Table 4:** Objective evaluation value of each alternative.

Alternative	$C_{1}$	$C_{2}$	$C_{3}$	$C_{4}$
A	(0.4,0.3)	(0.6,0.3)	(0.5,0.4)	(0.2,0.7)
B	(0.5,0.4)	(0.5,0.3)	(0.2,0.7)	(0.7,0.1)
C	(0.4,0.3)	(0.3,0.5)	(0.6,0.2)	(0.5,0.2)
D	(0.5,0.5)	(0.4,0.5)	(0.4,0.4)	(0.5,0.4)
E	(0.6,0.3)	(0.6,0.4)	(0.3,0.6)	(0.6,0.3)

**Table 5:** Comprehensive grey correlation coefficient of alternatives under different grey resolution coefficients based on completely unknown attribute weights.

Alternative	ξ=0.40	ξ=0.50	ξ=0.60	ξ=0.70	ξ=0.80	ξ=0.90	ξ=1.00
A	0.8372	0.8544	0.8681	0.8793	0.8887	0.8967	0.9037
B	0.6807	0.7133	0.7388	0.7596	0.7769	0.7917	0.8045
C	0.8488	0.8730	0.8906	0.9039	0.9143	0.9226	0.9295
D	0.9479	0.9575	0.9641	0.9689	0.9726	0.9755	0.9779
E	0.7697	0.7930	0.8114	0.8266	0.8393	0.8501	0.8595

**Table 6:** Attribute weight values under different grey resolution coefficients.

Alternative	ξ=0.40	ξ=0.50	ξ=0.60	ξ=0.70	ξ=0.80	ξ=0.90	ξ=1.00
$\omega_{1}$	0.30	0.30	0.30	0.30	0.30	0.30	0.30
$\omega_{2}$	0.18	0.18	0.18	0.18	0.18	0.18	0.18
$\omega_{3}$	0.28	0.28	0.28	0.28	0.28	0.28	0.28
$\omega_{4}$	0.24	0.24	0.24	0.24	0.24	0.24	0.24

**Table 7:** Comprehensive grey correlation coefficient of alternatives under different grey resolution coefficients based on known range of attribute weight.

Alternative	ξ=0.40	ξ=0.50	ξ=0.60	ξ=0.70	ξ=0.80	ξ=0.90	ξ=1.00
A	0.8341	0.8517	0.8656	0.8771	0.8867	0.8948	0.9019
B	0.6805	0.7131	0.7387	0.7595	0.7768	0.7916	0.8044
C	0.8473	0.8718	0.8895	0.9029	0.9134	0.9218	0.9288
D	0.9476	0.9573	0.9639	0.9688	0.9725	0.9754	0.9778
E	0.7695	0.7928	0.8113	0.8264	0.8392	0.8500	0.8594

**Table 8:** Change degree of comprehensive grey correlation coefficient of alternatives under fluctuation of grey resolution coefficient.

Alternative	Δθ (Unknown Weight)	Δθ (Weight Range Known)
A	0.0665	0.0678
B	0.1238	0.1239
C	0.0807	0.0815
D	0.0300	0.0302
E	0.0898	0.0899

**Table 9:** Ranking results under different methods.

Methods	Ranking Results
The traditional intuitionistic fuzzy distance	D≻E≻C≻A≻B
The intuitionistic fuzzy cross-entropy distance (unknown weight)	D≻C≻A≻E≻B
The intuitionistic fuzzy cross-entropy distance (weight range known)	D≻C≻A≻E≻B

## References

[1] Liang Y., Tu Y., Ju Y., Shen W. (2019). A multi-granularity proportional hesitant fuzzy linguistic TODIM method and its application to emergency decision making. Int. J. Disaster Risk Reduct..

[2] Gao J., Xu Z., Liang Z., Liao H. (2019). Expected consistency-based emergency decision making with incomplete probabilistic linguistic preference relations. Knowl. Based Syst..

[3] Nassereddine M., Azar A., Rajabzadeh A., Afsar A. (2019). Decision making application in collaborative emergency response: A new PROMETHEE preference function. Int. J. Disaster Risk Reduct..

[4] Zadeh L.A. (1965). Fuzzy sets. Inf. Control.

[5] Hu D., Jiang T., Yu X.C. (2020). Construction of non-convex fuzzy sets and its application. Neurocomputing.

[6] Garg H., Chen S.M. (2020). Multiattribute group decision making based on neutrality aggregation operators of q-rung orthopair fuzzy sets. Inf. Sci..

[7] Atanassov K.T. (1986). Intuitionistic fuzzy sets. Fuzzy Sets Syst..

[8] Atanassov K.T., Gargov G. (1989). Interval-valued intuitionistic fuzzy sets. Fuzzy Sets Syst..

[9] Krawczak M., Szkatuła G. (2020). On matching of intuitionistic fuzzy sets. Inf. Sci..

[10] Ngan R.T., Son L.H., Ali M., Tamir D.E., Rishe N.D., Kandel A. (2020). Representing complex intuitionistic fuzzy set by quaternion numbers and applications to decision making. Appl. Soft Comput. J..

[11] Arora J., Tushir M. (2020). An Enhanced Spatial Intuitionistic Fuzzy C-means Clustering for Image Segmentation. Procedia Comput. Sci..

[12] Wang F., Wan S.P. (2020). Possibility degree and divergence degree based method for interval-valued intuitionistic fuzzy multi-attribute group decision making. Exp. Syst. Appl..

[13] Liu P.S., Diao H.Y., Zou L., Deng A.S. (2020). Uncertain multi-attribute group decision making based on linguistic-valued intuitionistic fuzzy preference relations. Inf. Sci..

[14] Gao Y., Li D.S., Zhong H. (2020). A novel target threat assessment method based on three-way decisions under intuitionistic fuzzy multi-attribute decision making environment. Eng. Appl. Artif. Intell..

[15] Xu Z.S. (2007). Some similarity measures of intuitionistic fuzzy sets and their applications to multiple attribute decision making. Fuzzy Optim. Decis. Mak..

[16] Park J.H., Hwang J.H., Park W.J. (2013). Similarity measure on intuitionistic fuzzy sets. J. Cent. South Univ..

[17] Hu K., Li J. (2013). The entropy and similarity measure of interval valued intuitionistic fuzzy sets and their relationship. Int. J. Fuzzy Syst..

[18] Chen S.M., Tan J.M. (1994). Handling multicriteria fuzzy decision-making problems based on vague set theory. Fuzzy Sets Syst..

[19] Hong D.H., Choi C.H. (2000). Multicriteria fuzzy decision-making problems based on vague set theory. Fuzzy Sets Syst..

[20] Wu J., Huang H.B., Cao Q.W. (2013). Research on AHP with interval-valued intuitionistic fuzzy sets and its application in multi-criteria decision making problems. Appl. Math. Model..

[21] Keshavarzfarda R., Makui A. (2015). An IF-DEMATEL-AHP based on triangular intuitionistic fuzzy numbers. Decis. Sci. Lett..

[22] Chatterjee K., Kar B.M., Kar S. Strategic decisions using intuitionistic fuzzy VIKOR method for information system outsourcing. Proceedings of the 2013 International Symposium on Computational and Business Intelligence.

[23] Liao H.C., Xu Z.S. (2013). VIKOR-based method for hesitant fuzzy multi-criteria decision making. Fuzzy Optim. Decis. Mak..

[24] Wu M.C., Chen T.Y. (2011). The ELECTRE multicriteria analysis approach based on Atanassov’s intuitionistic fuzzy sets. Exp. Syst. Appl..

[25] Vahdani B., Mousavi S.M., Tavakkoli M.R. (2013). A new de-sign of the elimination and choice translating reality method for multi-criteria group decision-making in an intuitionistic fuzzy environment. Appl. Math. Model..

[26] Yu Z., Xu Z., Ma Y. (2013). Prioritized multi-criteria decision making based on the idea of PROMETHEE. Procedia Comput. Sci..

[27] Meng F. (2015). An approach to Antanassov’s interval-valued intuitionistic fuzzy multi-attribute decision making based on prospect theory. Int. J. Comput. Intell. Syst..

[28] Luo Y., Wei G. Multiple attribute decision making with intuitionistic fuzzy information and uncertain attribute weights using minimization of regret. Proceedings of the 2009 4th IEEE Conference on Industrial Electronics and Applications.

[29] Xu Y.J., Wen X.W., Zhang W.C. (2018). A two-stage consensus method for large-scale multi-attribute group decision making with an application to earthquake shelter selection. Comput. Ind. Eng..

[30] Xu X.H., Wang L.L., Chen X.H., Liu B.S. (2019). Large group emergency decision-making method with linguistic risk appetites based on criteria mining. Knowl. Based Syst..

[31] Li M.Y., Cao P.P. (2019). Extended TODIM method for multi-attribute risk decision making problems in emergency response. Comput. Ind. Eng..

[32] Xiong W.T., Van Gelder P.H.A.J.M., Yang K.W. (2020). A decision support method for design and operationalization of search and rescue in maritime emergency. Ocean Eng..

[33] Wang Z.G., Hao H., Gao F., Zhang Q., Zhang J., Zhou Y.J. (2019). Multi-attribute decision making on reverse logistics based on DEA-TOPSIS: A study of the Shanghai End-of-life vehicles industry. J. Clean. Prod..

[34] Wu Q., Wu P., Zhou L.G., Chen H.Y., Guan X.J. (2018). Some new Hamacher aggregation operators under single-valued neurotrophic 2-tuple linguistic environment and their applications to multi-attribute group decision making. Comput. Ind. Eng..

[35] Karimi H., Sadeghi-Dastaki M., Javan M. (2020). A fully fuzzy best–worst multi attribute decision making method with tri-angular fuzzy number: A case study of maintenance assessment in the hospitals. Appl. Soft Comput. J..

[36] Xu Z.S. (2007). Models for multiple attribute decision-making with intuitionistic fuzzy information, International Journal of Un-certainty. Fuzziness Knowl. Based Syst..

[37] Burillo P., Bustince H. (1996). Entropy on intuitionistic fuzzy sets and on interval-valued fuzzy sets. Fuzzy Sets Syst..

[38] Ye J. (2011). Fuzzy cross entropy of interval-valued intuitionistic fuzzy sets and its optimal decision-making method based on the weigths of alternatives. Exp. Syst. Appl..

[39] Ji Y.M., Qi M.L. (2020). A robust optimization approach for decontamination planning of emergency planning zone: Facility location and assignment plan. Socio Econ. Plan. Sci..

[40] Dey A., Zaman K. (2020). A robust optimization approach for solving two-person games under interval uncertainty. Comput. Oper. Res..

[41] Ji Y., Qu S.J., Wu Z., Liu Z.M. (2020). A Fuzzy-Robust Weighted Approach for Multicriteria Bilevel Games. IEEE Trans. Ind. Inf..

